# Identification and Typing of Human Enterovirus: A Genomic Barcode Approach

**DOI:** 10.1371/journal.pone.0026296

**Published:** 2011-10-14

**Authors:** Chengguo Wei, Guoqing Wang, Xin Chen, Honglan Huang, Bin Liu, Ying Xu, Fan Li

**Affiliations:** 1 Department of Pathogeny Biology, Norman Bethune Medical College of Jilin University, Changchun, Jilin, China; 2 Computational Systems Biology Laboratory, Department of Biochemistry and Molecular Biology, and Institute of Bioinformatics, University of Georgia, Athens, Georgia, United States of America; 3 College of Computer Science and Technology, Jilin University, Changchun, Jilin, China; 4 The First Bethune Hospital of Jilin University, Changchun, Jilin, China; Blood Systems Research Institute, United States of America

## Abstract

Identification and typing of human enterovirus (HEVs) are important to pathogen detection and therapy. Previous phylogeny-based typing methods are mainly based on multiple sequence alignments of specific genes in the HEVs, but the results are not stable with respect to different choices of genes. Here we report a novel method for identification and typing of HEVs based on information derived from their whole genomes. Specifically, we calculate the k-*mer* based barcode image for each genome, HEV or other human viruses, for a fixed k, 1<k<7, where a genome barcode is defined in terms of the k-*mer* frequency distribution across the whole genome for all combinations of k-*mers*. A phylogenetic tree is constructed using a barcode-based distance and a neighbor-joining method among a set of 443 representative non-HEV human viruses and 395 HEV sequences. The tree shows a clear separation of the HEV viruses from all the non-HEV viruses with 100% accuracy and a separation of the HEVs into four distinct clads with 93.4% consistency with a multiple sequence alignment-based phylogeny. Our detailed analyses of the HEVs having different typing results by the two methods indicate that our results are in better agreement with known information about the HEVs.

## Introduction

Human enterovirus (HEVs) are a genus of (+)ssRNA viruses, and they are among the most common human viruses, causing a wide range of acute diseases, such as upper respiratory tract infection, febrile rash, encephalitis, acute flaccid paralysis [1]and severe chronic disorders [2], [3], [4], [5]. The prevalence and the clinical significance of HEVs are further manifested by multiple outbreaks of the hand, foot and mouth disease (HFMD) in Asia, mainly caused by enterovirus 71 [6], [7], [8], [9]. As of now, over 100 serotypes of the HEV have been documented [10], [11], [12], [13], and only a handful of them can cause severe diseases [14] such as poliomyelitis by poliovirus [15]. It is known that 83.5% of the HEV-related disease cases were caused by 15 serotypes [16]. Therefore, classification of HEVs is important to designing novel diagnostic and treatment strategies.

A number of methods have been developed for classification (typing) of HEVs. The traditional method, based on biological properties of viruses such as antigenic differences [17], subdivided HEVs into poliovirus (PV), coxsackievirus (CV) groups A and B, echovirus, and the ‘new’ serotypes designated as EV-68 through EV-71 [18], [19], [20]. This method is expensive and time consuming, and could not handle some of the recently discovered HEV types such as some coxsackieviruses due to the lack of specific antisera [21]. Molecular techniques such as RT-PCR in conjunction with sequence alignment-based phylogeny reconstruction algorithms offer a sensitive and rapid alternative for the classification of HEVs. Based on a specific gene shared by the HEV genomes, this approach divides HEVs into four types: HEV-A through D [22]. However, this approach is not stable when tested using a different set of HEV genes, giving rise to a classification result (VP1 [23], [24], [25], VP2 [26], VP3 [27], VP4 [28]), inconsistent with the first classification result. Phylogeny reconstruction based on the whole HEV genomes will not work easily since these genomes are not well conserved in multiple aspects including the gene orders and different levels of conservation for different sets of orthologous genes. And many genetic fragment, emerging with metagenome sequencing technique development, can not be classified using this sequence alignment method.

We present a novel classification method based on information derived from the whole genome sequences of HEVs instead of specific genes. To the best of our knowledge, there has not been any published research on virus typing using information derived from the whole genome sequences. Instead of appending all the gene sequences from the HEVs and then building a phylogeny based on such artificial sequences, which could be highly sensitive to weighting factors for different genes, we use information more intrinsic to individual HEV genomes to construct the phylogeny. Specifically we have used a barcode representation to represent each HEV genome [29]. We have previously demonstrated that each organism has a unique barcode image; and more closely related genomes generally have more similar barcodes [29]. This provides the basis for our barcode-based phylogeny analysis.

The basic idea of the genomic barcode is to represent a genome using a two-dimensional array with the row representing the genome axis contracted by N fold, the column representing the axis of all k-*mers* for a fixed k (1<k<7), arranged in alphabetical order, and the value at row i and column j is the frequency of the i^th^ k-*mer* within the window from base-pair j*N + 1 to base-pair (j+1)*N, with N being the window size (the default values of the barcode program [29] are k = 4 and N = 1,000 base-pairs (bps) but can be adjusted by the user). One very interesting property of any genomic barcode is that the frequency distribution for any k-*mer* (for a fixed 1<k<7) is highly stable across the whole genome. Hence if the frequencies are mapped to gray levels with higher frequencies mapped to lighter gray levels, each column of the barcode representation gives rise to a line generally having a consistent gray level. Barcodes not only provide a good tool for visualizing genomes, but also allow easy comparisons between different genomes. One simple way to compare two genome barcodes is through compare their average frequencies over the whole genomes across the whole list of k-*mer* although more sophisticated ways can be used to capture more information of the targeted barcodes [29].

## Results and Discussion

### Genomic barcodes of human viruses

**Figure 1:**
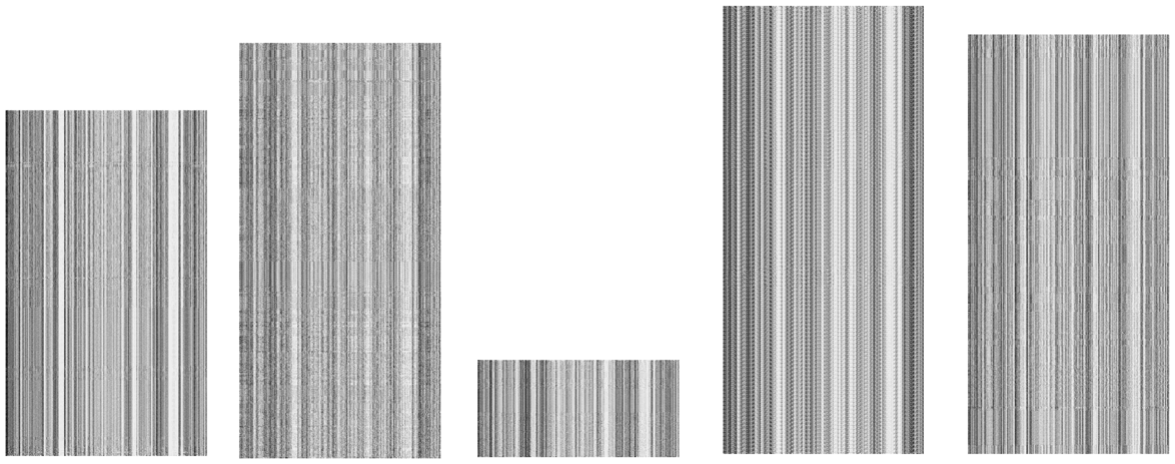
Barcodes of five representative human viruses: (a) HIV, (b) Enterovirus, (c) Rabies virus, (d) SARS Coronavirus, and (e) Hepatitis B virus. For each barcode, the x-axis is the list of all unique combinations of 4-mers arranged in the alphabetical order, the y-axis is same kind of virus joint genome axis contracted by 2,000 fold, and the gray level shows the frequency of each k-mer within a 2,000 bp window in the corresponding location.

We have calculated the barcodes for a total of 838 human ssRNA virus genomes of four families, namely HIV (279), Rabies virus (63), SARS coronavirus (101) and HEVs (395) using the barcode server at http://csbl1.bmb.uga.edu/Barcode/. We also calculated a barcode for dsDNA virus genome, Hepatitis B virus (993) as comparison. Figure 1 shows the barcodes of one representative genome for each of the five virus families, where different heights for different barcodes reflect sizes of the joint sequence of the same kind of virus. We can see from the barcode images that different viruses have different barcode images. Furthermore, it should be noted that regardless whether a virus is DNA (Hepatitis B virus) or RNA virus (HIV, Rabies virus, SARS coronavirus, HEVs) its genome has this barcode property. It's worth noting that, if we change the parameter N and k, the frequency of different k-*mers* were consistent.

### Typing of HEVs

**Figure 2:**
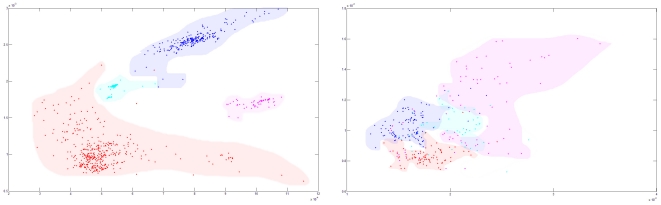
Identification and typing of HEVs. The x-axis for each plot is the distance between the feature vector of each virus' barcode and the average feature vector of all the viruses we used (in A we used the average feature vector of four kinds of virus: HIV, HEV, SARS and rabies virus; in B we used the average feature vector of all subtypes of HEV), and the y-axis is the distance between the feature vector of each virus' barcode and a normalization vector with value  = 1/136 for each of its dimensions, where 136 is the total of number of unique k-*mers* (paired with its reverse complement [32]). (A): the red dots represent HEVs (395 genomes), the blue ones for HIV (279 genomes), the magenta ones for SARS coronavirus (101 genomes), and the cyan ones for rabies virus genomes (63 genomes). (B): the blue dots represent poliovirus (78 genomes), the green ones for echovirus (52 genomes), the red ones for new virus strain enterovirus 68-71 (72 genomes), and the magenta ones for coxsackievirus A and B group genomes (85 genomes).

We have studied the barcode similarities among the 838 virus genomes from four families measured in terms of two specific distances, for k = 4 (see Figure 2 for definition and results). Figure 2A shows the scattering plot of all the 838 viruses in the two-dimensional space defined by the two distance measures. From the figure, we can see that the four families of viruses can be well separated (through non-linear functions) in this two dimensional space. In addition, we can also see the enterovirus have a relatively large variation measured by the current two features, compared to other families of viruses. The mechanism of HEVs having very great genetic diversity was not very clear yet. Some reports show that Polioviruses had greater genetic diversity duo to the frequent recombination [30]. Maybe these mechanism works in all HEVs.

We subsequently applied a similar method to all the HEVs. Figure 2B shows the scattering plot of the four different types of HEVs, which are color-coded based on the classification results by a phylogenic analysis using a specific gene of the HEVs. Although there was a small overlap between echovirus and coxakievirus, most of the HEV stains can be correctly clustered with clear boundaries, which shows the typing accuracy of this barcode approach on HEVs

### Comparing HEV typing results based on barcodes *versus* specific genes

**Figure 3:**
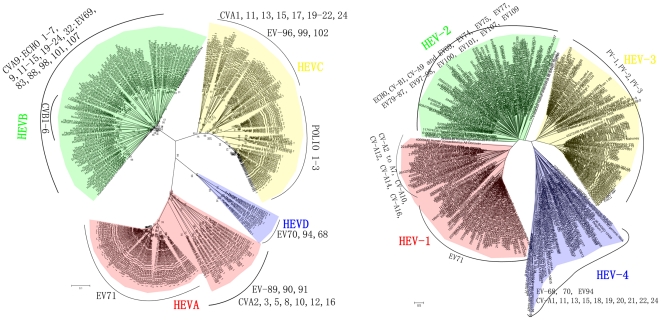
Phylogenetic trees for the HEVs based on a specific HEV gene (A) *versus* the HEV barcodes (B). The edge lengths in the trees reflect the genetic distance calculated according to the Kimura-parameter model. The VP1-based tree's reliability was estimated using 1,000 bootstrap replications. The serotype names beside the trees denote what the serotypes of the species having HEVs.

In order to analyze the accuracy of HEV typing results based on barcodes, we generate two phylogenetic trees for HEV. Figure 3A shows the phylogenetic tree we constructed based on the VP1 gene of HEVs using the MEGA Clustal-W alignment and neighbor joining clustering method [31], which groups the HEVs into four clads, named HEV-A through D. We also did a reconstruction of a phylogeny based on the barcodes of all the HEV genomes, as shown in Figure 3B, which also gives rise to four large clads, named HEV1 through 4. Clearly the two trees are largely in agreement except for some CV-A strains, giving rise to a consistency level at 93.4%. The details of the differences are given in Table 1. The difference between the two classification results is whether HEVs of serotype CVA1, 11, 13, 15, 18, 19, 20, 21, 22 or 24 should be grouped together with EV68, 70 and 94 or PV1, 2 and 3. The reason causing these differences between these two methods is that we can obtain some more information from a whole genome view. We have carried out extensive literature to find any previous reports that may suggest our classification method is reliable to have biological meaning. For instance, EV70 and CVA24 can both cause a highly contagious eye disease, acute hemorrhagic conjunctivitis [32], [33], [34]. The details of the differences are given in Table 1. The difference between the two classification results is whether HEVs of serotype CVA1, 11, 13, 15, 18, 19, 20, 21, 22 or 24 should be grouped together with EV68, 70 and 94 or PV1, 2 and 3. The reason causing these differences between these two methods is that we can obtain some more information from a whole genome view. We have carried out extensive literature to find any previous reports that may suggest our classification method is reliable to have biological meaning. For instance, EV70 and CVA24 can both cause a highly contagious eye disease, acute hemorrhagic conjunctivitis [32], [33], [34].

**Table 1:** Comparison of one gene-based and whole genome barcode-based phylogenetic trees (the numbers inside parentheses are the number of virus types for the corresponding serotype).

Num.	Typing results by two methods			
	Serotypes	Barcode based	One gene based	Comparison
1	EV71(82), CV-A2 to A7(1), CV-A10(1), CV-A12(1), CV-A14(1), CV-A16(3)	HEV-1	HEV-A	Exact match
2	CV-A9(1), CV-B1(1), CV-B2(3), CV-B3(15), CV-B4 to 6(3)E3(2), E4(3), E5(2), E6(3), E7(2), E9(4), E11(4), E12 to 16(1), E18 to 20(1), E24(1), E25(2), E26(1), E27(1), E29(1), E30(6), E30 to 33(1)EV69(1), EV74(1), EV75(1), EV77(1), EV79-87(1), EV97-98(2), EV100(2), EV101(1), EV107(2), EV109(1)	HEV-2	HEV-B	Exact match
3	PV-1(72), PV-2(32), PV-3(13)	HEV-3	HEV-C	Exact match
4	CV-A1(1),CV-A11(2),CV-A13(3), CV-A15(2), CV-A18(3), CV-A19(2), CV-A20(4), CV-A21(3), CV-A22(3), CV-A24(3)	HEV-4	HEV-C	**Different**
5	EV-68(2), 70(2), EV94(2)	HEV-4	HEV-D	Exact match
Match rate: 93.41%*				

*The match rate is calculated by the match sequence number/total sequence number.

In a word, with the next generation high-throughput sequencing technique developing (Solexa, SOLiD, et al.), sequencing a bacterial and virus genome is not challenging work. In the future, how to take advantage of this available whole genomic sequence information will give us new vision to identify viruses. Otherwise, we could find more and more metagenomic sequences have been generated so far, most of them are fragments without any VP1 gene sequences, our barcode-based method has the metagenome binning property, which can be found in our previous paper[29]. We hope this genome featured method could be wildly used as more and more whole genomic sequences have been generated.

### Concluding remark

Due to various factors such as the high diversity and the plasticity of the RNA genomes, accurate typing of HEVs remains a challenging task. The purpose of this work is to provide a new genome typing method,which allows utilizing information derived from whole genome sequences instead of specific genes. Since this method does not rely on detailed sequence information, it avoids the issue in finding the “correct” set of orthologous genes for phylogenetic analysis, which is particularly useful for virus genomes as they generally do not have signature genes like 16S rRNA gene for genomes of living organisms, making such whole-genome based phylogeny analysis particularly useful for viruses.

Through our study, we demonstrated that this new method is at least as good as the widely used specific gene-based phylogeny reconstruction even when using more sophisticated phylogeny reconstruction algorithms.

## Materials and Methods

### Virus sequence data

Five classes of viruses' complete genomes, HIV, human enterovirus, Rabies virus, SARS coronavirus and Hepatitis B virus are retrieved from (http://www.ncbi.nlm.nih.gov/nucleotide/) using Bioperl. The information of these five classes of viruses is given in Table 2.

**Table 2:** Information of five classes of viruses' complete genome sequences.

Virus	Genome Length	Genome Number
HIV	∼9,006	279
HEVs	∼7,200	395
Rabies virus	∼11,900	63
SARS Coronavirus	∼29,700	101
Hepatitis B virus	∼3,200	993

### Calculation of genomic barcode distance

We calculated the barcode using the genomic barcode server at http://csbl1.bmb.uga.edu/Barcode/nsertion.php. For each kind of virus, we firstly made the same kind viruses genomes head to tail into a long sequence, then partitioned it into non-overlapping fragments of M = 2,000bps long and calculated 4-*mer* based barcode for each genome. Specifically, the barcode for each genome is a matrix of K = 136 columns and genome_length/M rows, with the *i^th^* value being the combined frequency of the *i^th^* 4-*mer* and its reverse complement in this fragment. Actually, we obtained the HEVs' complementary strand by base pairing and calculated k-*mer* reverse complement frequency. We had proved that combines of a k-*mer* and its reverse complement are more reliable and accurate in classifying organisms in our previous work [29]. Then, we mapped the k-*mer* frequencies to grey levels so we can generate a barcode image for a whole genome (as well as for each segment of the genome), darker grey levels are for lower frequencies. The distance between two barcodes is calculated as the Euclidean distance between the corresponding 136-dimensional vectors. For two such matrices M1 and M2 with K columns and L rows, we defined their barcode distance as 
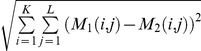
. For details, we refer the reader to [29].

### Phylogenetic trees building using barcode distance and Clustal W alignment

We calculated the barcode distance between two HEV genomes using the above barcode distance. Then we entered the pair-wise distance among all the genomes under consideration into the MEGA meg file to build the phylogenetic tree using neighbor-joining method in MEGA 4 software.
